# DNA repair phenotype and cancer risk: a systematic review and meta-analysis of 55 case–control studies

**DOI:** 10.1038/s41598-022-07256-7

**Published:** 2022-03-01

**Authors:** Hui-Chen Wu, Rebecca Kehm, Regina M. Santella, David J. Brenner, Mary Beth Terry

**Affiliations:** 1 Department of Environmental Health Sciences, Mailman School of Public Health of Columbia University, 630 West 168th St., Room P&S 16-421E, New York, NY 10032 USA; 2 Herbert Irving Comprehensive Cancer Center, Columbia University Medical Center, New York, NY USA; 3 Department of Epidemiology, Mailman School of Public Health of Columbia University, New York, NY USA; 4 Center for Radiological Research, Columbia University Irving Medical Center, 630W 168th Street, New York, NY 10032 USA

**Keywords:** Cancer, Cancer epidemiology

## Abstract

DNA repair phenotype can be measured in blood and may be a potential biomarker of cancer risk. We conducted a systematic review and meta-analysis of epidemiological studies of DNA repair phenotype and cancer through March 2021. We used random-effects models to calculate pooled odds ratios (ORs) of cancer risk for those with the lowest DNA repair capacity compared with those with the highest capacity. We included 55 case–control studies that evaluated 12 different cancers using 10 different DNA repair assays. The pooled OR of cancer risk (all cancer types combined) was 2.92 (95% Confidence Interval (CI) 2.49, 3.43) for the lowest DNA repair. Lower DNA repair was associated with all studied cancer types, and pooled ORs (95% CI) ranged from 2.02 (1.43, 2.85) for skin cancer to 7.60 (3.26, 17.72) for liver cancer. All assays, except the homologous recombination repair assay, showed statistically significant associations with cancer. The effect size ranged from 1.90 (1.00, 3.60) for the etoposide-induced double-strand break assay to 5.06 (3.67, 6.99) for the γ-H2AX assay. The consistency and strength of the associations support the use of these phenotypic biomarkers; however large-scale prospective studies will be important for understanding their use related to age and screening initiation.

## Introduction

Cancer initiation is classically associated with the induction of mutations in key oncogenes or tumor suppressor genes, due to the presence of unrepaired/misrepaired DNA lesions produced by endogenous or exogenous genotoxic agents^1^. Many risk factors for cancer such as smoking, ionizing radiation, and diet can induce DNA damage^2^. Higher levels of DNA/protein adducts in blood from exogenous exposures are associated with increased cancer risk^3^. DNA repair plays a fundamental role in the maintenance of genomic integrity^4^. Individuals with deficiency in DNA repair capacity might be more susceptible to cancer risk.

**Figure 1:**
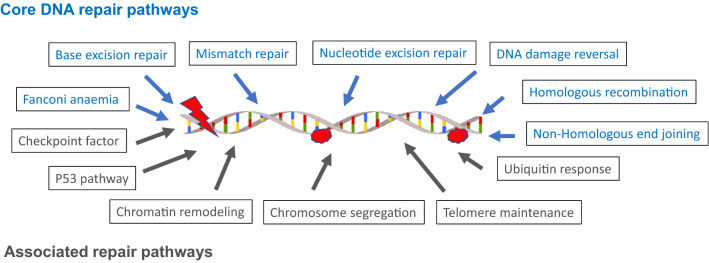
DNA repair pathways.

DNA repair capacity can be assessed either with genomic/proteomic approaches or with phenotypic approaches^5^. A concern with genomic/proteomic approaches is that mammalian DNA damage repair mechanisms are extraordinarily complex. In humans it involves ~ 450 genes in 13 different pathways including 7 core and 6 associated pathways, with over half the proteins interacting with other proteins from different pathways (Fig. 1)^6^; it follows that any specific genomic or proteomic methodology is unlikely to reflect overall DNA repair capacity. If it were possible to characterize the genetic complexity, it would be extremely challenging to implement at a clinical level. By contrast, phenotypic approaches—e.g., inducing DNA damage and then measuring the rate of DNA repair or the amount of unrepaired DNA damage, or both—have the potential to be more reflective of overall DNA repair capacity^7^. DNA repair phenotyping assays use fresh or cryopreserved peripheral blood mononuclear cells (PBMC) or lymphoblastoid cell lines as a surrogates for target tissue of DNA repair^7^. A phenotypic assay, if it is high throughput, may be more feasible to implement in a clinical setting as phenotypic approaches can reflect the totality of multiple complex pathways.

The purpose of our systematic review and meta-analysis is to quantitatively and qualitatively summarize the literature regarding DNA repair phenotype and risk of cancer. We assessed the association of DNA repair phenotype biomarkers with the risk of cancer by conducting a meta-analysis from all epidemiological studies published through March 2021.

## Results

### Overall summary of number and study design of studies

 Detailed characteristics of the included studies are shown in Supplemental Table 1. Based on the inclusion eligibility, we identified 55 studies of 12 different cancer types: lung (n = 20), breast (n = 10), skin (n = 7), head and neck (n = 7), bladder (n = 2), esophageal (n = 2), upper aerodigestive tract (n = 2), prostate (n = 1), gastric (n = 1), colorectal (n = 1), gliomas (n = 1) and liver (n = 1). All studies used a case–control study design and most used blood collected at the time of cancer diagnosis; only two studies were nested case–control studies using blood collected before cancer diagnosis^8,9^. The first nested-case control study was by Sigurdson et al.^8^ and used three DNA repair assays: comet assay, host cell reactivation assay and mutagen sensitivity assay in blood collected between 0.3 and 6 years before lung cancer^8^. The authors reported an OR of 2.09 (95% CI 1.00, 4.37) for lung cancer risk among individuals at the highest quartile of chromatid breaks/cell compared with individuals at the lowest quartile measured by the mutagen sensitivity assay. The ORs were 1.2 (95% CI 0.54, 2.65) for the comet assay and 0.96 (0.45, 2.04) for the host cell reactivation assay. The second nested case–control design was by Shen et al.^9^ and used a modified host cell reactivation assay to measured homologous recombination repair capacity in bloods collected from 152 breast cancer patients and their matched controls and reported an OR of 1.42 (95% CI 1.21, 2.52). A similar magnitude effect size was then found in the validation set of 50 cases-control pairs using blood collected before cancer diagnosis^9^.

**Figure 2:**
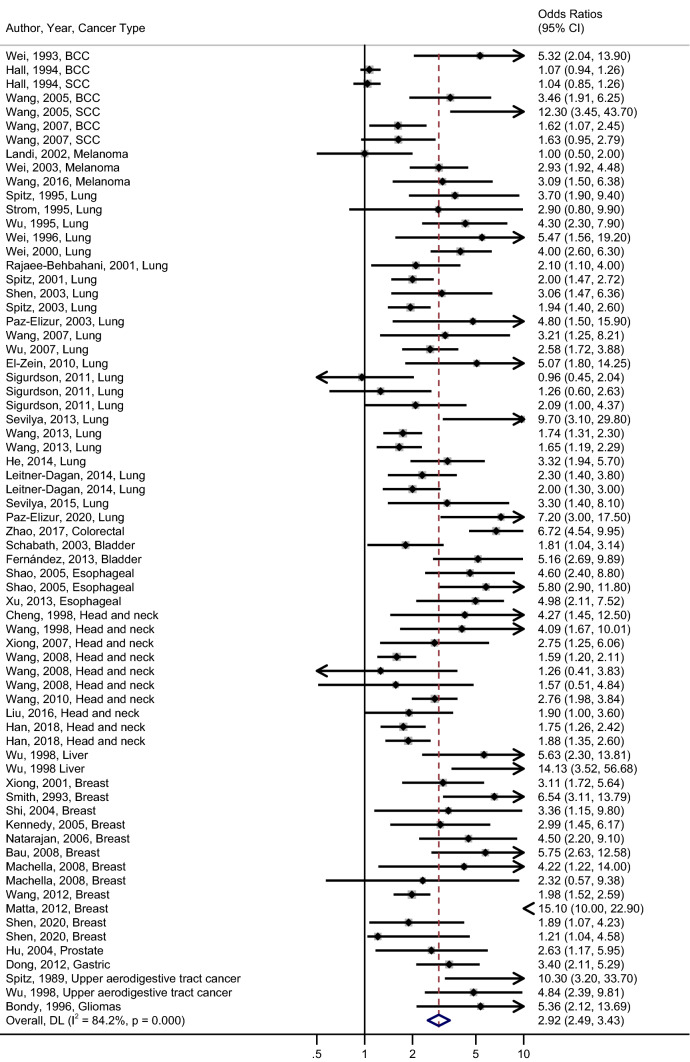
Forest plot of meta-analysis of lower DNA repair capacity and cancer risk in the random effect model. Individual studies are represented by ORs and 95% CI. The dashed line indicates the value of the overall pooled OR.

The overall pooled OR (95% CI) for DNA repair deficiency and cancer risk was 2.92 (2.49, 3.43) (Fig. 2). We saw significant heterogeneity across different studies (*I*^*2*^ = 84.2%; *p*-value from Cochran’s Q < 0.0001), and the Funnel plot suggested possible publication bias (*p*-value from Egger’s Test < 0.0001; see Supplemental Fig. 1). We further looked by cancer type and assay to better understand the sources of heterogeneity.

### Cancer type

**Figure 3:**
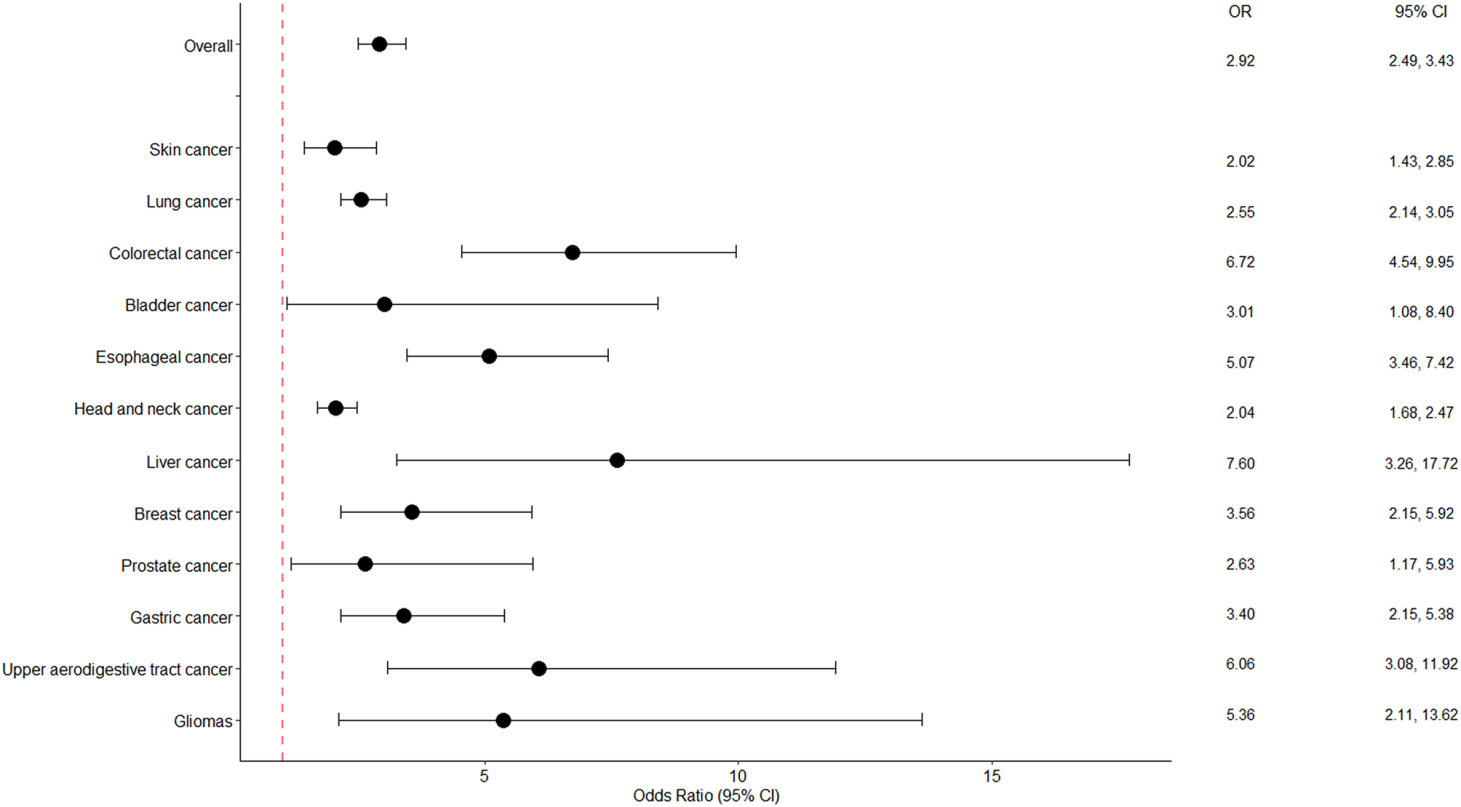
Forest plot of meta-analysis of lower DNA repair capacity and cancer risk by cancer type in the random effect model. Individual cancers are represented by ORs and 95% CI. The dashed line indicates the value of 1.

 We found lower DNA repair phenotype was associated with all studied cancer types, and the pooled ORs ranged from 2.02 (1.43, 2.85) for skin cancer to 7.60 (3.26, 17.72) for liver cancer (Supplemental Fig. 2, and Fig. 3). We observed heterogeneity across skin, lung, bladder, and breast cancer studies, while there was no evidence of heterogeneity across studies for esophageal, head and neck, or upper aerodigestive tract cancers.

### Assay type

**Figure 4:**
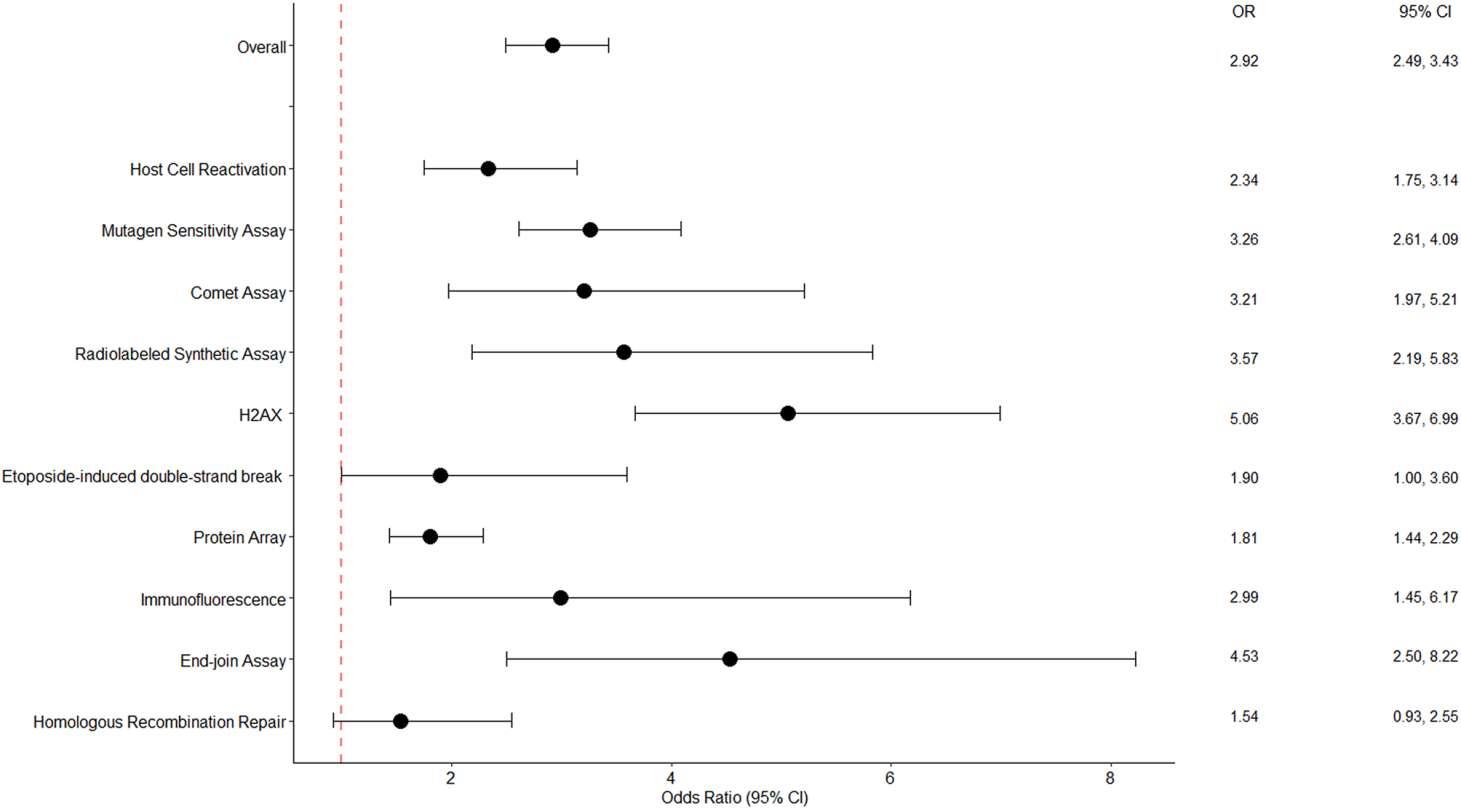
Forest plot of meta-analysis of lower DNA repair capacity and cancer risk by assay type in the random effect model. Individual assays are represented by ORs and 95% CI. The dashed line indicates the value of 1.

 In our meta-analysis, there were 10 DNA repair phenotyping assays including the host-cell reactivation (n = 18), mutagen sensitivity (n = 18), comet (n = 6), radiolabeled synthetic (n = 5), γ-H2AX (n = 4), end-joining (n = 2), etoposide (ETOP)-induced double strand break (n = 1), nucleotide excision repair protein (n = 1), homologous recombination repair, (n = 1), and immunofluorescence assays (n = 1). The pooled ORs (95% CI) were 2.34 (1.75, 3.14) for the host-cell reactivation assay, 3.26 (1.75, 3.14) for the mutagen sensitivity assay, 3.21 (1.97, 5.21) for the comet assay, 5.06 (3.67, 6.99) for the γ-H2AX assay (Supplemental Fig. 3 and Fig. 4). Studies using the host-cell reactivation, mutagen sensitivity, comet, and radiolabeled synthetic assay had evidence of heterogeneity across studies.

We further examined the association of DNA repair deficiency by assay type among lung and breast cancer studies, the- most frequent studies and common cancers. We found the effects of lower DNA repair capacity for lung cancer risk were similar across the different assay types (range of ORs = 2.14, 3.57) (Supplemental Fig. 4A). Although there was heterogeneity across studies within assay groups, we did not see statistically significant heterogeneity in the ORs for lung cancer pooled across assay groups (*p* = 0.21). We did observe statistically significant heterogeneity across different assays in the breast cancer studies (*p* = 0.01), where the host cell reactivation assay showed the largest effect size with a pooled OR of 7.75 (1.79, 33.49) (Supplemental Fig. 4B).

## Discussion

The meta-analysis we conducted summarized data from 55 studies and supported the hypothesis that individuals with lower DNA repair capacity are at increased susceptibility to cancer development and this result was consistent across cancer types and specific DNA repair phenotypic assays. This finding suggests that measuring DNA repair phenotype can potentially identify high-risk individuals for effective primary prevention, and for risk-based screening options.

Accurately identifying high-risk individuals is essential for effective primary prevention (e.g., chemoprevention)^10^, and for risk-based screening options^11^ which emphasize risk rather than age for optimal screening outcomes. Cancer risk prediction models incorporating minimally invasive blood markers including genetic variants^12^ and epigenetic markers^13^ have shown modest improvement in discriminatory accuracy. The magnitude of the associations between DNA repair phenotype and cancer risk is much stronger compared with the effect size measured by genetic variants of DNA repair genes (ORs range from 1 to 2)^14^. Our previous study examined the association of DNA double strand break repair capacity with breast cancer risk^15^. We found the largest differences in the DNA repair capacity between cases and controls were observed in women younger than 40 years^15^. Cancer risk models incorporating DNA repair phenotypic markers may significantly improve current cancer risk prediction^16^. However, to potentially integrate DNA repair phenotyping data into risk assessment, more studies are needed to examine intra-individual variability in DNA repair phenotyping over time, to assess whether a single measure at the time of first breast screening is useful or whether multiple measures over time are needed.

In our meta-analysis, we found there was significant heterogeneity across studies, which might be related to different cancer types and different DNA repair phenotyping assays. A potential explanation for why we observed heterogeneity across studies of different cancer types and assays might be related to the complex interplay of genetic and environmental factors in most cancer types. Analysis of the mutation burden of 27 tumor types found that there is substantial inter-individual variation in tumor mutational burden between cancer types and within individual tumor types^17^. Moreover, variability of the cell-based assays related to inter-laboratory experimental protocols is a challenge for inference^5, 18^. Differences in the experimental conditions including dose of the DNA damage reagents, cell types and cell culture condition might contribute to the heterogeneity across different studies^18^. It is known that different lymphocyte subsets response to DNA damage differentially; stimulated and non-stimulated lymphocytes also behave differently after DNA damage^19–21^. To better interpret the heterogeneity across different studies, cancer types and assays, future studies should report procedures and results following OECD guidelines^22^. In addition, we observed a potential publication bias, suggesting studies with statistically significant effects were more likely to be published.

Most studies use PBMC as surrogate tissues, assuming that PBMC are a legitimate surrogate for DNA repair in other tissues. The correlation between DNA repair capacity between target and blood is limited to one study that found a good correlation between OGG activity in blood and lung tissues from the same individual^23^. Although assays using blood samples are more feasible to implement in a clinical setting, more studies are needs to evaluate the correlation of DNA repair phenotype between blood and target tissues using different assays. There are numerous methods for measuring DNA repair directly, and each has its strengths and weaknesses^24^. Most of the assays such as the host-cell reactivation, mutagen sensitivity, immunofluorescence assays measure nucleotide excision repair capacity^25^. Nucleotide excision repair eliminates a wide variety of different forms of DNA damage and especially deals with bulky DNA damage/adducts induced by chemical carcinogens and dimers induced by ultraviolet light^26^. Methods such as the comet, host-cell reactivation and radiolabeled synthetic assays can potentially measure different DNA repair pathways. End-joining, homologous recombination repair and γH2AX assays focus on repair of double strand breaks. In our analysis, we found the estimated effect sizes were consistent and of high magnitude across different assays and pathways. However, DNA repair functions are redundant in the context of cellular DNA damage, and there are back-up systems. If one of the critical DNA repair pathways is impaired, other pathways may be activated complicating understanding risk^27^.

Functional DNA repair assays are fundamentally more powerful than genotyping. But currently, there are few DNA repair assays available for epidemiologic studies because the assays are labor and time intensive. Thus studies to date are limited and there are no large-scale prospective studies or high-throughput phenotypic assays^28^. The resultant lack of population studies integrating these potentially informative measures with other factors limits our understanding of the fundamental cellular response to environmental exposures. However, recently our group developed a high-throughput γ-H2AX assay based on imaging flow cytometry (IFC) which is a faster and more efficient technique for assessing global double strand break repair capacity^29^. This IFC-based γ-H2AX protocol may provide a practical and high-throughput platform for measurements of individual global DNA double strand break repair capacity which can facilitate precision medicine by predicting individual radiosensitivity and risk of developing adverse effects related to radiotherapy treatment. The blood drop method of analysis of γH2AX is a simple and fast assay for large scale studies, screening and routine biomonitoring of exposure^30^. Cancer susceptibility is inherently complex, and polygenetic risk scores using genetic data have been established and show improvement in prediction accuracy for cancer^31^. Our meta-analysis supports a strong association between global repair capacity and cancer risk. Measuring DNA repair capacity is a potentially powerful marker to identify subgroups at high risk of cancer. Measuring overall DNA repair capacity markers in blood may be one way of understanding the role of DNA damage and repair in cancer risk and might provide intermediate outcome markers in prevention studies. Measuring DNA repair capacity may provide a potentially robust method to identify individuals that can benefit from individual-based health risk assessment and personalized risk reduction strategies. Established high-throughput measurement of DNA repair phenotyping may also be more feasible to implement in a clinic setting as opposed to complex genomic and proteomic approaches. Incorporating DNA repair phenotype into risk models may improve model discriminatory accuracy but will need large-scale prospective evidence to understand the role of timing and age at measurement and cancer screening initiation.

## Materials and methods

We used the following MeSH terms in our literature search: “cancer” AND “DNA repair phenotype” OR “DNA repair capacity” OR “comet assay” OR “Host-cell reactivation” OR “γ-H2AX assay” OR “Mutagen sensitivity assay” for studies published from 1980 to 20 March 2021 (Supplemental Fig. 5). Our initial search of the PubMed database restricted to studies that were conducted in humans and published in the English language returned 2045 publications for further screening. We first reviewed the title and abstract of each study and excluded 1932 studies that (1) did not examine cancer as an outcome, (2) did not use a cellular assay for DNA damage and repair, and (3) did not compare differences in DNA damage and repair between cancer cases and unaffected controls using either case–control or cohort study designs. We then reviewed the remaining 113 studies and restricted our analysis to studies (n = 55) that estimated effect size of DNA damage and repair between cancer cases and unaffected controls. We searched the reference lists of the included publications for additional eligible publications, but no additional studies were identified. The remaining 55 publications were included in our review^8, 9, 15, 32–83^. We extracted data on study population, study design, sample size, DNA repair phenotyping assay, confounding assessment, and effect estimates for the group with the lowest DNA repair capacity compared with the group with the highest capacity, and the corresponding 95% confidence intervals (CIs) from the included publications. When a study reported results on different racial groups or different damage reagent, we treated each group as a separate comparison in our meta-analysis. Studies included in the current meta-analysis had to meet both of the following criteria: (1) use an epidemiological study design such as a case–control or cohort study design, and (2) present odds ratios or rate ratios.

### Statistical analysis

We conducted a meta-analysis to calculate pooled estimated odds ratio (ORs) across studies using random-effects models to account for between study heterogeneity. To assess the heterogeneity among studies, we used the Cochran Q test^84^ and I squared (I^2^) statistics^85^. Cochran Q test is calculated as the weighted sum of squared differences between individual study effects and the pooled effect across studies. The I^2^ statistics describes the percentage of variation across studies that is due to heterogeneity rather than chance. We used a funnel plot^86^ to assess the risk of bias and examine metal-analysis validity. In the absence of bias, studies are symmetrically distributed around the fixed effect size estimate, due to sampling error being random. When bias is present, study-level effects will be asymmetrically distributed around the global fixed-effect estimate.

To examine possible publication bias, we generated funnel plots and used the Egger’s test^87^ to examine if there were small study effects. We also used an influence plot to evaluate if individual studies were impacting overall summary estimates. We performed subgroup analyses stratified by the tumor site and assay type. We only report results from the random-effects models, and not fixed-effects models, as we found there was significant heterogeneity across the different studies. Analyses were performed using the software Stata 15.1 (College Station, TX)^88^. All *P*-values were two-sided.

## Supplementary Information

Supplementary Figure 1.

Supplementary Figure 2.

Supplementary Figure 3.

Supplementary Figure 4A.

Supplementary Figure 4B.

Supplementary Information.

## Data Availability

Contact the corresponding author for any inquiries regarding data or analytical code.

## References

[1] Poirier MC, Santella RM, Weston A (2000). Carcinogen macromolecular adducts and their measurement. Carcinogenesis.

[2] Phillips DH, Venitt S (2012). DNA and protein adducts in human tissues resulting from exposure to tobacco smoke. Int. J. Cancer..

[3] Shen J, Liao Y, Hopper JL, Goldberg M, Santella RM, Terry MB (2017). Dependence of cancer risk from environmental exposures on underlying genetic susceptibility: An illustration with polycyclic aromatic hydrocarbons and breast cancer. Br. J. Cancer..

[4] Hoeijmakers JHJ (2001). Genome maintenance mechanisms for preventing cancer. Nature.

[5] Berwick M, Vineis P (2000). Markers of DNA repair and susceptibility to cancer in humans: An epidemiologic review. JNCI: J. Natl. Cancer Inst..

[6] Pearl LH, Schierz AC, Ward SE, Al-Lazikani B, Pearl FMG (2015). Therapeutic opportunities within the DNA damage response. Nat. Rev. Cance..

[7] Nagel ZD, Engelward BP, Brenner DJ (2017). Towards precision prevention: Technologies for identifying healthy individuals with high risk of disease. Mutat. Res./Fundam. Mol. Mech. Mutagenes..

[8] Sigurdson AJ, Jones IM, Wei Q (2011). Prospective analysis of DNA damage and repair markers of lung cancer risk from the Prostate, Lung, Colorectal and Ovarian (PLCO) Cancer Screening Trial. Carcinogenesis.

[9] Shen J, Song R, Chow W-H, Zhao H (2020). Homologous recombination repair capacity in peripheral blood lymphocytes and breast cancer risk. Carcinogenesis.

[10] Padamsee TJ, Wills CE, Yee LD, Paskett ED (2017). Decision making for breast cancer prevention among women at elevated risk. Breast Cancer Res..

[11] Lee CI, Chen LE, Elmore JG (2017). Risk-based breast cancer screening: Implications of breast density. Med. Clin. N. Am..

[12] Wacholder S, Hartge P, Prentice R (2010). Performance of common genetic variants in breast-cancer risk models. N. Engl. J. Med..

[13] Xu Z, Bolick SC, DeRoo LA, Weinberg CR, Sandler DP, Taylor JA (2013). Epigenome-wide association study of breast cancer using prospectively collected sister study samples. J. Natl. Cancer Inst..

[14] Roberts MR, Shields PG, Ambrosone CB (2011). Single-nucleotide polymorphisms in DNA repair genes and association with breast cancer risk in the web study. Carcinogenesis.

[15] Machella N, Terry MB, Zipprich J (2008). Double-strand breaks repair in lymphoblastoid cell lines from sisters discordant for breast cancer from the New York site of the BCFR. Carcinogenesis.

[16] Paz-Elizur T, Leitner-Dagan Y, Meyer KB (2019). DNA repair biomarker for lung cancer risk and its correlation with airway cells gene expression. JNCI Cancer Spectrum..

[17] Lawrence MS, Stojanov P, Polak P (2013). Mutational heterogeneity in cancer and the search for new cancer-associated genes. Nature.

[18] Collins AR, El Yamani N, Lorenzo Y, Shaposhnikov S, Brunborg G, Azqueta A (2014). Controlling variation in the comet assay. Front. Genet..

[19] Heylmann D, Ponath V, Kindler T, Kaina B (2021). Comparison of DNA repair and radiosensitivity of different blood cell populations. Sci. Rep..

[20] Hu Q, Xie Y, Ge Y, Nie X, Tao J, Zhao Y (2018). Resting T cells are hypersensitive to DNA damage due to defective DNA repair pathway. Cell Death Dis..

[21] Heylmann D, Badura J, Becker H, Fahrer J, Kaina B (2018). Sensitivity of CD3/CD28-stimulated versus non-stimulated lymphocytes to ionizing radiation and genotoxic anticancer drugs: Key role of ATM in the differential radiation response. Cell Death Dis..

[22] Møller P, Azqueta A, Boutet-Robinet E (2020). Minimum Information for Reporting on the Comet Assay (MIRCA): Recommendations for describing comet assay procedures and results. Nat. Protoc..

[23] Paz-Elizur T, Krupsky M, Blumenstein S, Elinger D, Schechtman E, Livneh Z (2003). DNA repair activity for oxidative damage and risk of lung cancer. JNCI: J. Natl. Cancer Inst..

[24] Nagel ZD, Chaim IA, Samson LD (2014). Inter-individual variation in DNA repair capacity: A need for multi-pathway functional assays to promote translational DNA repair research. DNA Repair.

[25] Li C, Wang L-E, Wei Q (2009). DNA repair phenotype and cancer susceptibility—A mini review. Int. J. Cancer.

[26] Schärer OD (2013). Nucleotide excision repair in eukaryotes. Cold Spring Harb. Perspect. Biol..

[27] Yoshimoto K, Mizoguchi M, Hata N (2012). Complex DNA repair pathways as possible therapeutic targets to overcome temozolomide resistance in glioblastoma. Front. Oncol..

[28] Nagel ZD, Kitange GJ, Gupta SK (2017). DNA repair capacity in multiple pathways predicts chemoresistance in glioblastoma multiforme. Can. Res..

[29] Lee Y, Wang Q, Shuryak I, Brenner DJ, Turner HC (2019). Development of a high-throughput γ-H2AX assay based on imaging flow cytometry. Radiat. Oncol..

[30] Heylmann D, Kaina B (2016). The γH2AX DNA damage assay from a drop of blood. Sci. Rep..

[31] Kachuri L, Graff RE, Smith-Byrne K (2020). Pan-cancer analysis demonstrates that integrating polygenic risk scores with modifiable risk factors improves risk prediction. Nat. Commun..

[32] Wei Q, Matanoski GM, Farmer ER, Hedayati MA, Grossman L (1993). DNA repair and aging in basal cell carcinoma: a molecular epidemiology study. Proc. Natl. Acad. Sci. U.S.A..

[33] Hall J, English DR, Artuso M, Armstrong BK, Winter M (1994). DNA repair capacity as a risk factor for non-melanocytic skin cancer—A molecular epidemiological study. Int. J. Cancer.

[34] Landi MT, Baccarelli A, Tarone RE (2002). DNA repair, dysplastic nevi, and sunlight sensitivity in the development of cutaneous malignant melanoma. J. Natl. Cancer Inst..

[35] Wei Q, Lee JE, Gershenwald JE (2003). Repair of UV light-induced DNA damage and risk of cutaneous malignant melanoma. J. Natl. Cancer Inst..

[36] Wang L-E, Xiong P, Strom SS (2005). In vitro sensitivity to ultraviolet B light and skin cancer risk: A case–control analysis. JNCI: J. Natl. Cancer Inst..

[37] Wang LE, Li C, Strom SS (2007). Repair capacity for UV light induced DNA damage associated with risk of nonmelanoma skin cancer and tumor progression. Clin. Cancer Res..

[38] Wang LE, Li C, Xiong P (2016). 4-nitroquinoline-1-oxide-induced mutagen sensitivity and risk of cutaneous melanoma: A case-control analysis. Melanoma Res..

[39] Spitz MR, Hsu TC, Wu X, Fueger JJ, Amos CI, Roth JA (1995). Mutagen sensitivity as a biological marker of lung cancer risk in African Americans. Cancer Epidemiol. Biomark. Prev..

[40] Strom SS, Wu S, Sigurdson AJ (1995). Lung cancer, smoking patterns, and mutagen sensitivity in Mexican-Americans. J. Natl. Cancer Inst. Monogr..

[41] Wu X, Delclos GL, Annegers JF (1995). A case-control study of wood dust exposure, mutagen sensitivity, and lung cancer risk. Cancer Epidemiol. Biomark. Prev..

[42] Wei Q, Cheng L, Hong WK, Spitz MR (1996). Reduced DNA repair capacity in lung cancer patients. Cancer Res..

[43] Wei Q, Cheng L, Amos CI (2000). Repair of tobacco carcinogen-induced DNA adducts and lung cancer risk: a molecular epidemiologic study. J. Natl. Cancer Inst..

[44] Rajaee-Behbahani N, Schmezer P, Risch A (2001). Altered DNA repair capacity and bleomycin sensitivity as risk markers for non-small cell lung cancer. Int. J. Cancer.

[45] Spitz MR, Wu X, Wang Y (2001). Modulation of nucleotide excision repair capacity by XPD polymorphisms in lung cancer patients. Cancer Res..

[46] Shen H, Spitz MR, Qiao Y (2003). Smoking, DNA repair capacity and risk of nonsmall cell lung cancer. Int. J. Cancer.

[47] Spitz MR, Wei Q, Dong Q, Amos CI, Wu X (2003). Genetic susceptibility to lung cancer: the role of DNA damage and repair. Cancer Epidemiol. Biomark. Prev..

[48] Paz-Elizur T, Krupsky M, Blumenstein S, Elinger D, Schechtman E, Livneh Z (2003). DNA repair activity for oxidative damage and risk of lung cancer. J. Natl. Cancer Inst..

[49] Wang L, Wei Q, Shi Q, Guo Z, Qiao Y, Spitz MR (2007). A modified host-cell reactivation assay to measure repair of alkylating DNA damage for assessing risk of lung adenocarcinoma. Carcinogenesis.

[50] Wu X, Lin J, Etzel CJ (2007). Interplay between mutagen sensitivity and epidemiological factors in modulatinglung cancer risk. Int. J. Cancer.

[51] El-Zein RA, Monroy CM, Cortes A, Spitz MR, Greisinger A, Etzel CJ (2010). Rapid method for determination of DNA repair capacity in human peripheral blood lymphocytes amongst smokers. BMC Cancer.

[52] Sevilya Z, Leitner-Dagan Y, Pinchev M (2014). Low integrated DNA repair score and lung cancer risk. Cancer Prev. Res. (Phila)..

[53] Wang LE, Gorlova OY, Ying J (2013). Genome-wide association study reveals novel genetic determinants of DNA repair capacity in lung cancer. Cancer Res..

[54] He Y, Gong Y, Lin J (2013). Ionizing radiation-induced γ-H2AX activity in whole blood culture and the risk of lung cancer. Cancer Epidemiol. Biomark. Prev..

[55] Leitner-Dagan Y, Sevilya Z, Pinchev M (2014). Enzymatic MPG DNA repair assays for two different oxidative DNA lesions reveal associations with increased lung cancer risk. Carcinogenesis.

[56] Sevilya Z, Leitner-Dagan Y, Pinchev M (2015). Development of APE1 enzymatic DNA repair assays: Low APE1 activity is associated with increase lung cancer risk. Carcinogenesis.

[57] Paz-Elizur T, Leitner-Dagan Y, Meyer KB (2020). DNA repair biomarker for lung cancer risk and its correlation with airway cells gene expression. JNCI Cancer Spectr..

[58] Zhao L, Chang DW, Gong Y, Eng C, Wu X (2017). Measurement of DNA damage in peripheral blood by the γ-H2AX assay as predictor of colorectal cancer risk. DNA Repair (Amst.)..

[59] Schabath MB, Spitz MR, Grossman HB (2003). Genetic instability in bladder cancer assessed by the comet assay. J. Natl. Cancer Inst..

[60] Fernández MI, Gong Y, Ye Y (2013). γ-H2AX level in peripheral blood lymphocytes as a risk predictor for bladder cancer. Carcinogenesis.

[61] Shao L, Lin J, Huang M, Ajani JA, Wu X (2005). Predictors of esophageal cancer risk: Assessment of susceptibility to DNA damage using comet assay. Genes Chromosomes Cancer..

[62] Xu E, Gong Y, Gu J, Jie L, Ajani JA, Wu X (2013). Risk assessment of esophageal adenocarcinoma using γ-H2AX assay. Cancer Epidemiol. Biomark. Prev..

[63] Cheng L, Eicher SA, Guo Z, Hong WK, Spitz MR, Wei Q (1998). Reduced DNA repair capacity in head and neck cancer patients. Cancer Epidemiol. Biomark. Prev..

[64] Wang LE, Sturgis EM, Eicher SA, Spitz MR, Hong WK, Wei Q (1998). Mutagen sensitivity to benzo(a)pyrene diol epoxide and the risk of squamous cell carcinoma of the head and neck. Clin. Cancer Res..

[65] Xiong P, Hu Z, Li C (2007). In vitro benzo[a]pyrene diol epoxide-induced DNA damage and chromosomal aberrations in primary lymphocytes, smoking, and risk of squamous cell carcinoma of the head and neck. Int. J. Cancer..

[66] Wang L-E, Xiong P, Zhao H, Spitz MR, Sturgis EM, Wei Q (2008). Chromosome instability and risk of squamous cell carcinomas of head and neck. Can. Res..

[67] Wang L-E, Hu Z, Sturgis EM (2010). Reduced DNA repair capacity for removing tobacco carcinogen-induced DNA adducts contributes to risk of head and neck cancer but not tumor characteristics. Clin. Cancer Res..

[68] Liu Z, Liu H, Gao F, Dahlstrom KR, Sturgis EM, Wei Q (2016). Reduced DNA double-strand break repair capacity and risk of squamous cell carcinoma of the head and neck—A case-control study. DNA Repair.

[69] Han P, Liu H, Shi Q (2018). Associations between expression levels of nucleotide excision repair proteins in lymphoblastoid cells and risk of squamous cell carcinoma of the head and neck. Mol. Carcinog..

[70] Wu X, Gu J, Patt Y (1998). Mutagen sensitivity as a susceptibility marker for human hepatocellular carcinoma. Cancer Epidemiol. Biomark. Prev..

[71] Xiong P, Bondy ML, Li D (2001). Sensitivity to benzo(a)pyrene diol-epoxide associated with risk of breast cancer in young women and modulation by glutathione S-transferase polymorphisms: a case-control study. Cancer Res..

[72] Smith TR, Miller MS, Lohman KK, Case LD, Hu JJ (2003). DNA damage and breast cancer risk. Carcinogenesis.

[73] Shi Q, Wang LE, Bondy ML, Brewster A, Singletary SE, Wei Q (2004). Reduced DNA repair of benzo[a]pyrene diol epoxide-induced adducts and common XPD polymorphisms in breast cancer patients. Carcinogenesis.

[74] Kennedy DO, Agrawal M, Shen J (2005). DNA repair capacity of lymphoblastoid cell lines from sisters discordant for breast cancer. J Natl Cancer Inst..

[75] Natarajan TG, Ganesan N, Carter-Nolan P, Tucker CA, Shields PG, Adams-Campbell LL (2006). γ-Radiation-induced chromosomal mutagen sensitivity is associated with breast cancer risk in African-American Women: Caffeine modulates the outcome of mutagen sensitivity assay. Cancer Epidemiol. Biomark. Prev..

[76] Bau D-T, Mau Y-C, Ding S-l, Wu P-E, Shen C-Y (2007). DNA double-strand break repair capacity and risk of breast cancer. Carcinogenesis.

[77] Wang LE, Han CH, Xiong P (2012). Gamma-ray-induced mutagen sensitivity and risk of sporadic breast cancer in young women: A case-control study. Breast Cancer Res. Treat..

[78] Hu JJ, Hall MC, Grossman L (2004). Deficient nucleotide excision repair capacity enhances human prostate cancer risk. Cancer Res..

[79] Dong H, Jin X, Hu J (2012). High γ-radiation sensitivity is associated with increased gastric cancer risk in a Chinese Han population: A case-control analysis. PLoS ONE.

[80] Spitz MR, Fueger JJ, Beddingfield NA (1989). Chromosome sensitivity to bleomycin-induced mutagenesis, an independent risk factor for upper aerodigestive tract cancers. Cancer Res..

[81] Wu X, Gu J, Hong WK (1998). Benzo[a]pyrene diol epoxide and bleomycin sensitivity and susceptibility to cancer of upper aerodigestive tract. J. Natl. Cancer Inst..

[82] Bondy ML, Kyritsis AP, Gu J (1996). Mutagen sensitivity and risk of gliomas: A case-control analysis. Cancer Res..

[83] Matta J, Echenique M, Negron E (2012). The association of DNA Repair with breast cancer risk in women. A comparative observational study. BMC Cancer.

[84] Higgins JP, Thompson SG (2002). Quantifying heterogeneity in a meta-analysis. Stat. Med..

[85] Higgins JP, Thompson SG, Deeks JJ, Altman DG (2003). Measuring inconsistency in meta-analyses. BMJ.

[86] Mikolajewicz N, Komarova SV (2019). Meta-analytic methodology for basic research: A practical guide. Front Physiol..

[87] Egger M, Smith GD, Schneider M, Minder C (1997). Bias in meta-analysis detected by a simple, graphical test. BMJ.

[88] StataCorp (2017). Stata Statistical Software: Release 15.

