# Supplementary figures and images for: DNA repair phenotype and cancer risk: a systematic review and meta-analysis of 55 case–control studies
Source: Sci Rep. 2022 Mar 1;12:3405. doi: 10.1038/s41598-022-07256-7 (PMC8888613; doi:10.1038/s41598-022-07256-7)

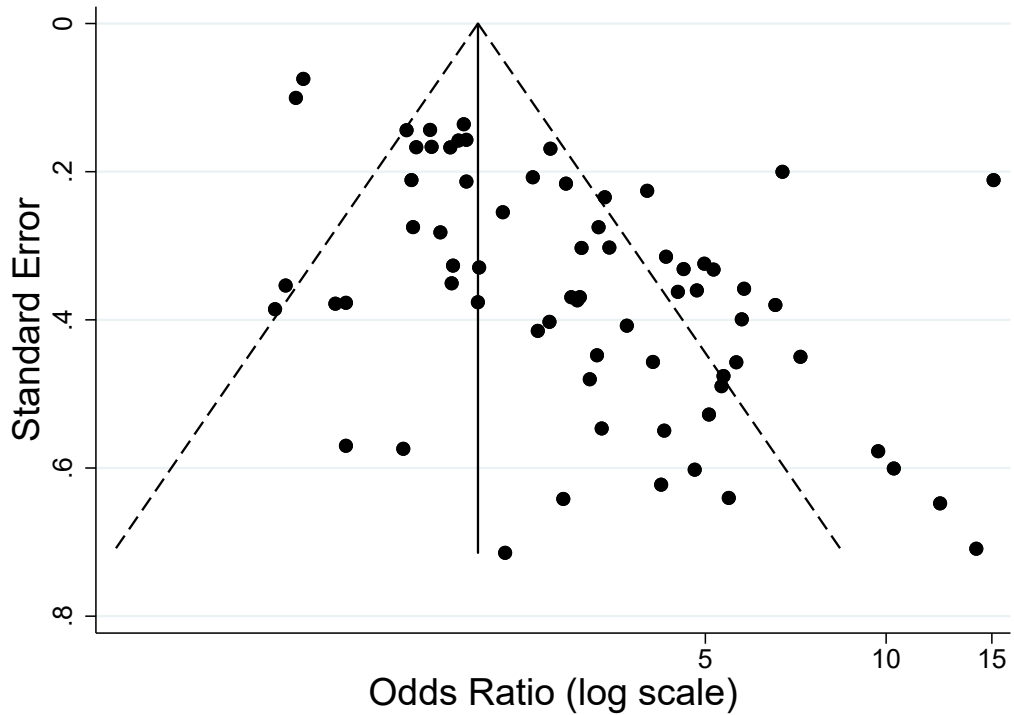

Supplement: Supplementary file 1 — Supplementary Figure 1. [file 41598_2022_7256_MOESM1_ESM.pdf]

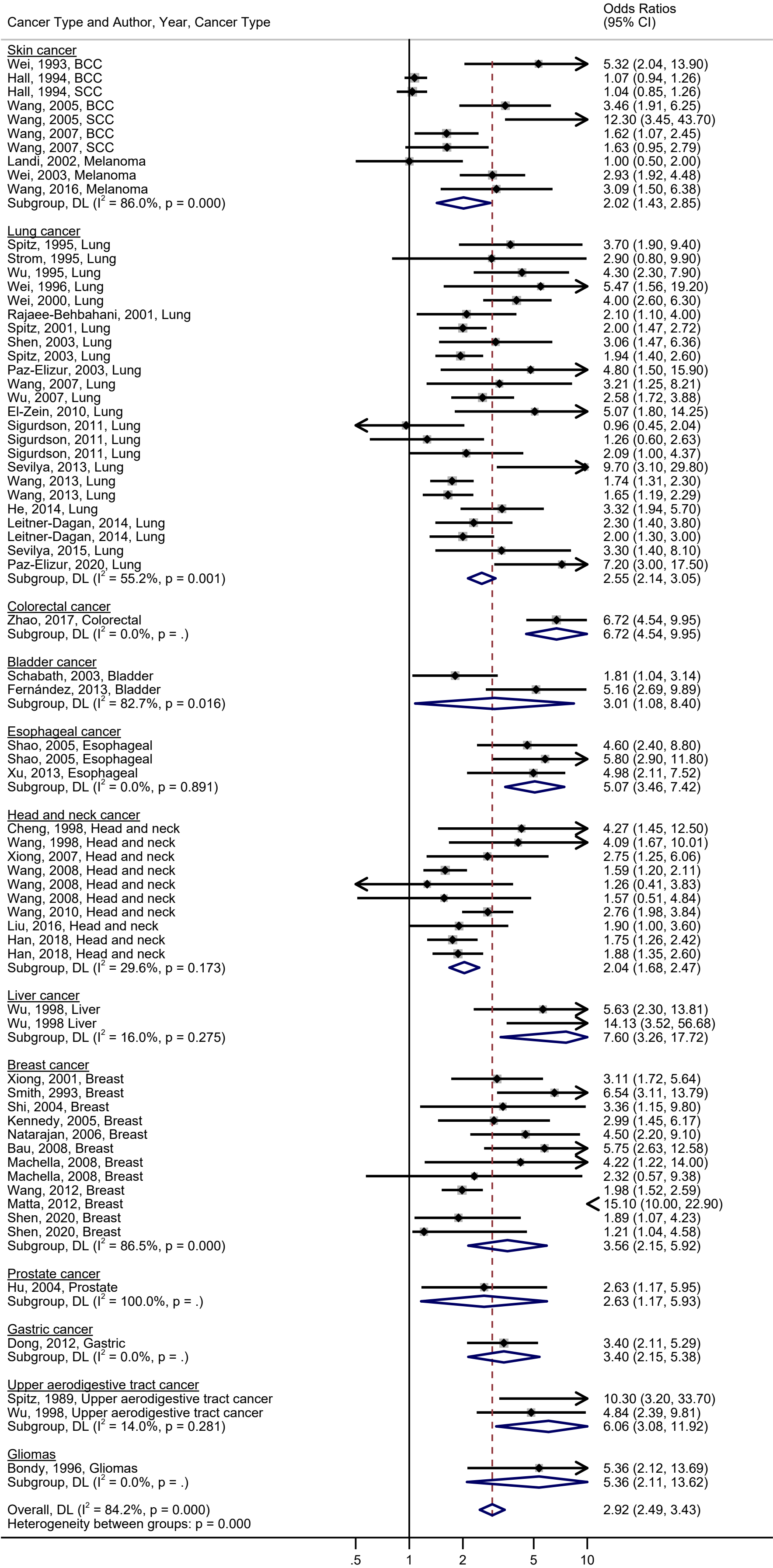

Supplement: Supplementary file 2 — Supplementary Figure 2. [file 41598_2022_7256_MOESM2_ESM.pdf]

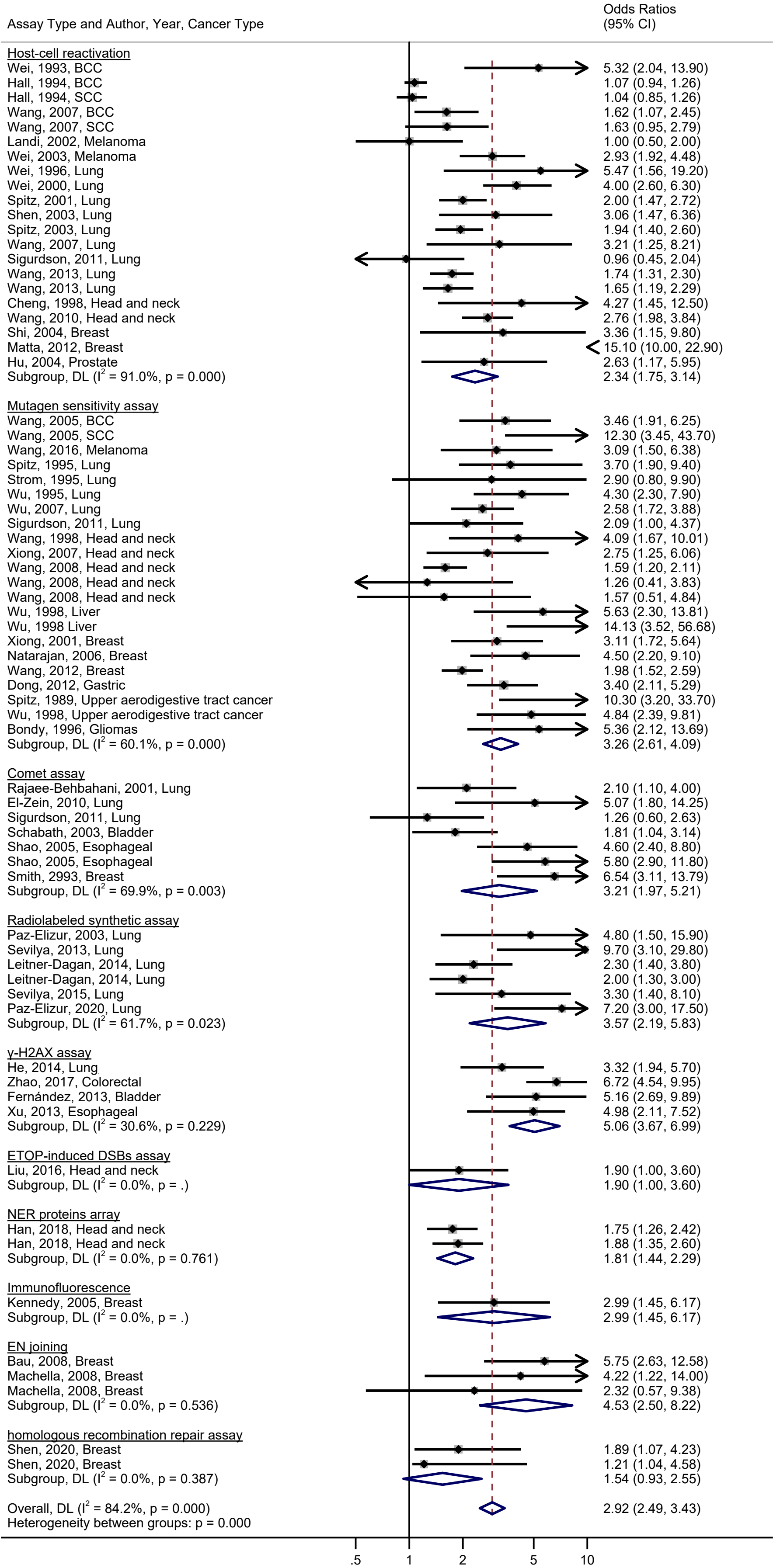

Supplement: Supplementary file 3 — Supplementary Figure 3. [file 41598_2022_7256_MOESM3_ESM.pdf]

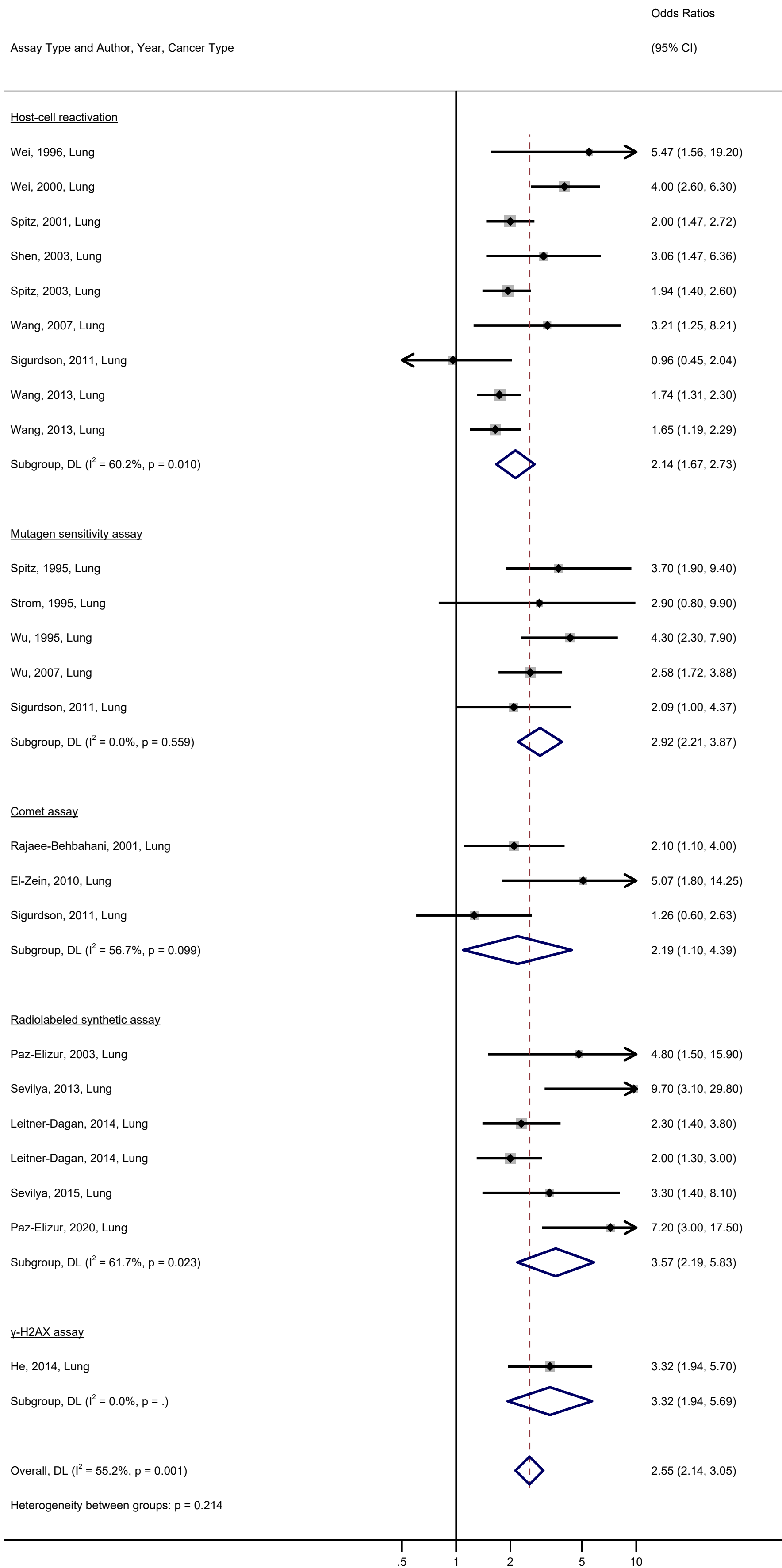

Supplement: Supplementary file 4 — Supplementary Figure 4A. [file 41598_2022_7256_MOESM4_ESM.pdf]

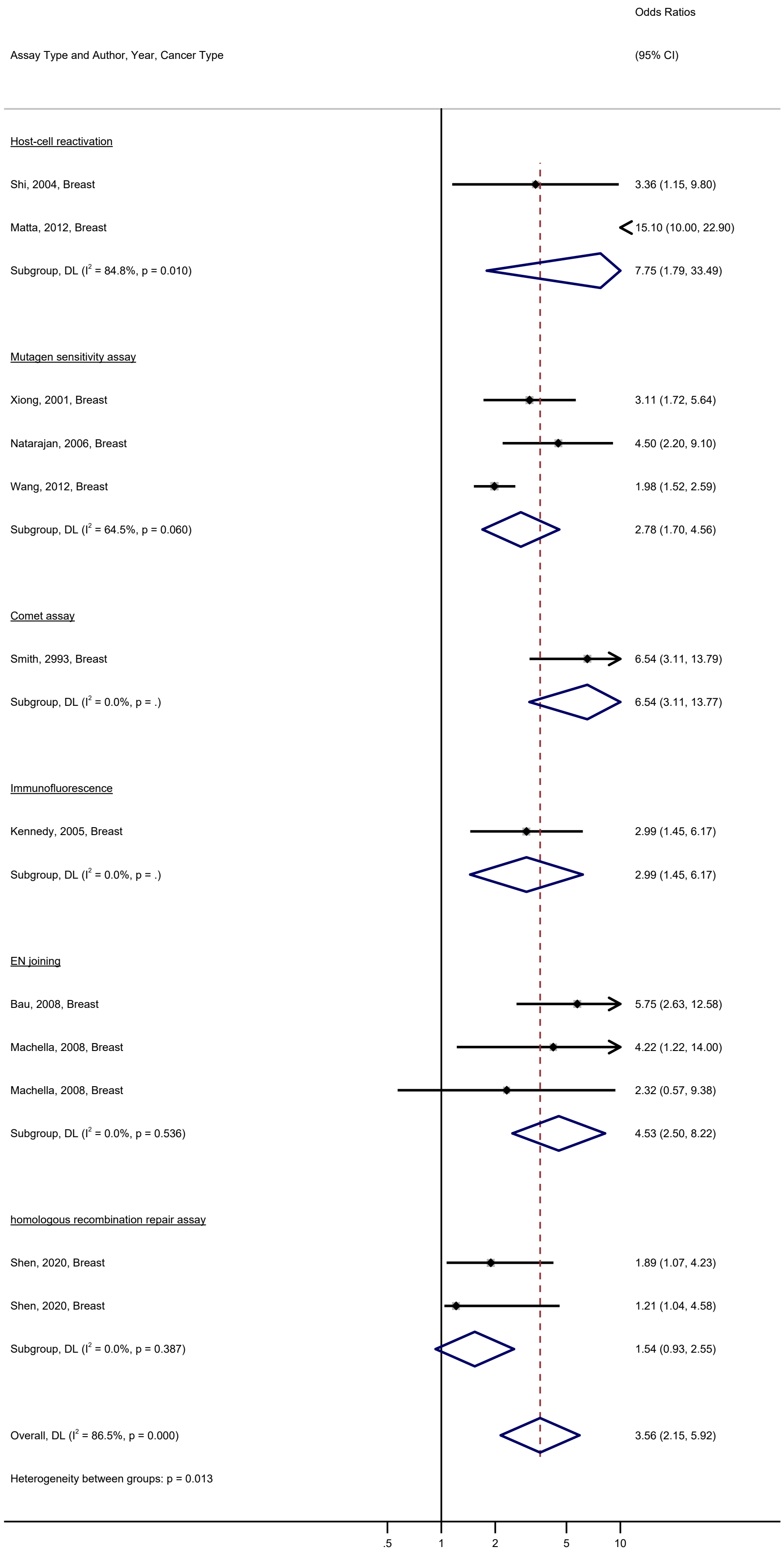

Supplement: Supplementary file 5 — Supplementary Figure 4B. [file 41598_2022_7256_MOESM5_ESM.pdf]
