# Supplementary material for: DNA repair phenotype and cancer risk: a systematic review and meta-analysis of 55 case–control studies
Source: Sci Rep. 2022 Mar 1;12:3405. doi: 10.1038/s41598-022-07256-7 (PMC8888613; doi:10.1038/s41598-022-07256-7)
Supplement: Supplementary file 6 — Supplementary Information. [file 41598_2022_7256_MOESM6_ESM.docx]

**DNA repair phenotype and cancer risk: a systematic review and meta-analysis of 55 case-control studies**

**Supplemental Figure 1**. **Funnel plot of DNA repair capacity and cancer risk in the meta-analysis**

**Supplemental Figure 2**. **Forest plot of meta-analysis of lower DNA repair capacity and cancer risk** **by cancer type in the random effect model. Individual studies are represented by ORs and 95% CI. The dashed line indicates the value of the overall pooled OR.**

**Supplemental Figure 3**. **Forest plot of meta-analysis of lower DNA repair capacity and cancer risk by assay type in the random effect model. Individual studies are represented by ORs and 95% CI. The dashed line indicates the value of the overall pooled OR.**

**Figure 4A. Forest plot of meta-analysis of lower DNA repair capacity and lung cancer risk by assay type in the random effect model. Individual studies are represented by ORs and 95% CI. The dashed line indicates the value of the overall pooled OR.**

**Figure 4B. Forest plot of meta-analysis of lower DNA repair capacity and breast cancer risk by assay type in the random effect model. Individual studies are represented by ORs and 95% CI. The dashed line indicates the value of the overall pooled OR.**

**Supplemental Figure 5**. **Flow chart of study selection**

PubMed search on key words: “cancer” AND “DNA repair phenotype” OR “DNA repair capacity” OR “comet assay” OR “Host-cell reactivation” OR “γ-H2AX assay” OR “Mutagen sensitivity assay”

(n=2045)

Excluded studies (n=1932) :1) examined cancer prognosis as an outcome, 2) did not have cellular based assay for DNA damage and repair, and 3) did not compare differences in DNA damage and repair between cancer cases and unaffected controls using either case-control or cohort study design.

Eligibility studies for meta-analysis (n=55)

Full-text articles assessed for eligibility (n=113)

Excluded studies (n=58) did not estimate effect size of DNA damage and repair between cancer cases and unaffected controls.

**Supplemental Table 1**. **Characteristics of the studies reporting on the association between DNA repair capacity and cancer risk**

| Author | Year | Country | Population | Assay | DNA Damage Reagent | Adjustment Factors |
| --- | --- | --- | --- | --- | --- | --- |
| Wei^1^ | 1993 | USA | 88 cases diagnosed with BCC, and 135 cancer-free controls. All subjects were 20-60 yr of age | Host-cell reactivation-CAT | UVC (254nm at 350 and 700 J/m2) | age, and sex |
| Hall^2^ | 1994 | Australia | 86 cases diagnosed with either BCC or SCC, and 87 controls. All subjects were 44-68 yr of age | Host-cell reactivation-CAT | UVC (254nm at 350 J/m2) | age, sex, and lymphocyte viability |
| Landi^3^ | 2002 | Italian | 132 cases diagnosed with melanoma and 145 age-, sex-matched controls | Host-cell reactivation-CAT | UVC (254nm at 350 J/m2) | age, sex, lymphocyte viability and sample storage time |
| Wei^4^ | 2003 | USA | 312 cases diagnosed with melanoma and 324 controls frequency-mated by age, and sex. | Host-cell reactivation-CAT | UVC (254 nm at 800 J/m2) | age, and sex |
| Wang^5^ | 2005 | USA | Cases diagnosed with either BCC (N=143) or SCC (N=88), and 327 controls. | Mutagen Sensitivity Assay | UVB (302nm at 150 J/m2) | age, and sex |
| Wang^6^ | 2007 | USA | Cases diagnosed with either BCC (N=146) or SCC (N=109), and 333 controls. | Host-cell reactivation-CAT | UVC (254nm at 800 J/m2) | age, sex, and sample storage time |
| Wang^7^ | 2016 | USA | 133 cases diagnosed with melanoma and 176 controls | Mutagen Sensitivity Assay | Nitroquinoline-1-oxide (10 µmol) | age, and sex |
| Spitz^8^ | 1995 | USA | 90 cases diagnosed with lung cancer and 119 controls. | Mutagen Sensitivity Assay | Bleomycin (0.03 unit/ml) | age, sex, smoking, education, and household size |
| Storm^9^ | 1995 | USA | 67 cases diagnosed with lung cancer and 107 controls. | Mutagen Sensitivity Assay | Bleomycin (0.03 unit/ml) | age, sex, and pack-year of smoking |
| Wu^10^ | 1995 | USA | 180 lung cancer cases), and 270 controls | Mutagen Sensitivity Assay | Colcemid (0.04 µg/ml) | age, sex, race, pack-year of smoking and overall wood dust exposure |
| Wei^11^ | 1996 | USA | 51 lung cancer cases, and 56 controls | Host-cell reactivation-CAT | Benzo(a)pyrene diol epoxide (75 µM) | age, sex, race, and smoking status. |
| Wei^12^ | 2000 | USA | 316 lung cancer cases, and 316 controls matched on age, sex and smoking status | Host-cell reactivation-CAT | Benzo(a)pyrene diol epoxide (60 µM) | age, sex, cell storage time, pack-year smoking, and family history of cancer |
| Rajaee-Behbahani^13^ | 2001 | Germany | 160 lung cancer cases, and 180 controls | Comet assay | Bleomycin (20 µg/ml) | age, and sex |
| Spitz^14^ | 2001 | USA | 341 lung cancer cases, and 360 age-, sex-, race- and smoking-matched controls | Host-cell reactivation-CAT | Benzo(a)pyrene diol epoxide (75 µM) |  |
| Shen^15^ | 2003 | USA | 467 lung cancer cases, and 488 controls | Host-cell reactivation-CAT | Benzo(a)pyrene diol epoxide (60 µM) | age, sex, race and pack-year of smoking |
| Spitz^16^ | 2003 | USA | 764 lung cancer cases, and 677 controls | Host-cell reactivation-CAT | Benzo(a)pyrene diol epoxide (60 µM) | age, sex and smoking status |
| Paz-Elizur^17^ | 2003 | Israel | 68 lung cancer cases, and 68 controls | Radiolabeled synthetic: OGG1 activity |  | smoking |
| Wang^18^ | 2007 | USA | 48 lung cancer cases, and 45 controls | Host-cell reactivation-luciferase | Nicotine-derived nitrosamine 4-(methylnitrosamino)-1-(3-pyridyl)-I-butanone | age, sex and smoking status |
| Wu^19^ | 2007 | USA | 977 lung cancer cases, and 977 controls | Mutagen Sensitivity Assay | Benzo(a)pyrene diol epoxide (2 µM) | age, sex and smoking status |
| El-Zein^20^ | 2010 | USA | 30 lung cancer cases, and 90 controls | Comet assay | H2O2 | age, sex, and family history of cancer |
| Sigurdson*^21^ | 2011 | USA | 117 lung cancer cases, and 117 controls | Host-cell reactivation-luciferase, comet assay, mutagen sensitive assay | Benzo(a)pyrene diol epoxide (60 µM) | age |
| Sevilya^50^ | 2013 | Israel | 96 lung cancer cases, and 96 controls | Radiolabeled synthetic: OGG1, MPG, APE1 |  |  |
| Wang^22^ | 2013 | USA | Discovery phase: 914 lung cancer cases, and 860 controls; Replication: 679 lung cancer cases, and 695 controls | Host-cell reactivation-CAT | Benzo(a)pyrene diol epoxide | age, sex, race and pack-year of smoking |
| He^52^ | 2014 | USA | 306 lung cancer cases, and 306 controls | γ-H2AX | Ionizing radiation (2.5 Gy) | age, sex, race, smoking status, dust exposure and emphysema |
| Leitner-Dagan^23^ | 2014 | Israel | 96 lung cancer cases, and 96 controls | Radiolabeled synthetic: OGG1, MPG |  | smoking |
| Sevilya^24^ | 2015 | Israel | 96 lung cancer cases, and 96 controls | Radiolabeled synthetic: APE |  | age, sex and smoking status |
| Paz-Elizur^25^ | 2020 | Israel | 150 lung cancer cases, and 143 controls | Radiolabeled synthetic: OGG1, MPG, APE1 |  | age, sex and smoking status |
| Zhao^26^ | 2017 | USA | 320 colorectal cancer cases, and 320 controls | γ-H2AX assay | Ionizing radiation (2.5 Gy) | age, sex and smoking status |
| Schabath^27^ | 2003 | USA | 114 bladder cancer cases, and 145 controls | Comet assay. | Gamma-radiation | age, sex, race and smoking status |
| Fernández^28^ | 2013 | USA | 174 bladder cancer cases, and 174 controls | γ-H2AX assay | Ionizing radiation (2.5 Gy) | age, sex, smoking status |
| Shao^29^ | 2005 | USA | 102 esophageal cancer cases, and 112 controls | Comet assay. | Benzo(a)pyrene diol epoxide and gamma-radiation | age, sex, race and smoking status |
| Xu^30^ | 2013 | USA | 211 esophageal cancer cases, and 211 controls | γ-H2AX assay | Ionizing radiation (2.5 Gy) | age, sex and smoking status |
| Cheng^31^ | 1998 | USA | 55 head and neck cancer cases, and 61 controls | Host-cell reactivation-CAT | Benzo(a)pyrene diol epoxide | age, sex, race, smoking status, and alcohol use |
| Wang^32^ | 1998 | USA | 60 head and neck cancer cases, and 112 controls | Mutagen Sensitivity Assay | Benzo(a)pyrene diol epoxide (4µM) | age, sex race, smoking status and alcohol drinking |
| Xiong^33^ | 2007 | USA | 123 head and neck cancer cases, and 136 controls | Comet assay. | Benzo(a)pyrene diol epoxide (4µM) | age, sex, smoking status and alcohol drinking |
| Wang^34^ | 2008 | USA | 895 head and neck cancer cases and 898 controls | Mutagen Sensitivity Assay | Benzo(a)pyrene diol epoxide (4µM) | age, sex, smoking and alcohol drinking |
| Wang^35^ | 2010 | USA | 744 head and neck cancer cases, and 753 controls | Host-cell reactivation-CAT | Benzo(a)pyrene diol epoxide (60µM) | age, sex, race, and smoking |
| Liu^36^ | 2016 | USA | 100 head and neck cancer cases, and 124 controls. | ETOP-induced DSBs assay | ETOP | age, sex, smoking and alcohol drinking |
| Han^37^ | 2018 | USA | 349 head and neck cancer cases, and 295 controls. | Nucleotide Excision Repair Proteins array |  | age, sex, smoking and alcohol drinking |
| Wu^38^ | 1998 | USA | 28 liver cancer, and 110 controls. | Mutagen Sensitivity Assay | Benzo(a)pyrene diol epoxide and Bleomycin |  |
| Xiong^39^ | 2001 | USA | 100 breast cancer cases, and 105 controls. | Mutagen Sensitivity Assay | Benzo(a)pyrene diol epoxide (4µM) | age, race, smoking and alcohol use |
| Smith^40^ | 2003 | USA | 70 breast cancer cases, and 70 controls. | Comet assay | Gamma-irradiation (6-Gy) | age |
| Shi^41^ | 2004 | USA | 69 breast cancer cases, and 79 controls. | Host-cell reactivation-CAT | Benzo(a)pyrene diol epoxide (60µM) | age, smoking, cell storage time, baseline CAT expression |
| Kennedy^42^ | 2005 | USA | 154 breast cancer cases, and 154 controls. | Immunofluorescence | Benzo(a)pyrene diol epoxide | age, BMI, and smoking |
| Natarajan^43^ | 2006 | USA | 61 breast cancer cases, and 84 controls. | Mutagen Sensitivity Assay | Gamma-irradiation (6-Gy) |  |
| Bau^44^ | 2007 | Taiwan | 112 breast cancer cases, and 108 controls. | End-joining assay |  | age, family history of cancer, BMI, and reproductive risk |
| Machella^45^ | 2008 | USA | 86 breast cancer cases, and 96 controls. | End- joining assay with EcoRI or HincII cut plasmid substrates |  | age, BMI, and smoking |
| Wang^46^ | 2012 | USA | 515 breast cancer cases, and 402 controls. | Mutagen sensitivity | Gamma-irradiation | age, smoking and alcohol use |
| Matta^47^ | 2012 | Puerto Rico | 285 breast cancer cases, and 539 controls. | Host-cell reactivation-luciferase | UVC (254nm at 350 and 700 J/m2) | age, BMI, family history of cancer, number of children, marital status and smoking |
| Shen*^48^ | 2020 | USA | 152 breast cancer cases, and 152 controls; Nested case-control study: 50 breast cancer cases, and 50 controls; | Homologous Recombination Repair assay |  | age, BMI, smoking, alcohol and physical activity |
| Hu^49^ | 2004 | USA | 140 prostate cancer cases, and 96 controls. | Host-cell reactivation-luciferase | UVC (254nm at 700 J/m2) | age, race, smoking status, and family history |
| Dong^50^ | 2012 | China | 517 gastric cancer cases, and 525 controls. | Mutagen Sensitivity Assay | Gamma-irradiation (1.5-Gy) | age, sex, H pylori infection, smoking and drinking |
| Spitz^51^ | 1989 | USA | 75 upper aerodigestive tract cancer cases, and 62 controls. | Mutagen Sensitivity Assay | Bleomycin |  |
| Wu^52^ | 1998 | USA | 67 upper aerodigestive tract cancer cases, and 81 controls. | Mutagen Sensitivity Assay | Benzo(a)pyrene diol epoxide | age, sex, ethnicity and smoking |
| Bondy^53^ | 1996 | USA | 45 Gliomas cancer cases, and 117 controls. | Mutagen Sensitivity Assay | Gamma-irradiation |  |

BCC: basal cell carcinoma; SCC: squamous cell carcinoma; CMM: cutaneous malignant melanoma; ETOP: etoposide

*blood were collected before cancer diagnosis

References

1. Wei Q, Matanoski GM, Farmer ER, Hedayati MA, Grossman L. DNA repair and aging in basal cell carcinoma: a molecular epidemiology study. *Proc Natl Acad Sci U S A.* 1993;90(4):1614-1618.PMC45925

2. Hall J, English DR, Artuso M, Armstrong BK, Winter M. DNA repair capacity as a risk factor for non-melanocytic skin cancer—a molecular epidemiological study. *International Journal of Cancer.* 1994;58(2):179-184

3. Landi MT, Baccarelli A, Tarone RE, et al. DNA repair, dysplastic nevi, and sunlight sensitivity in the development of cutaneous malignant melanoma. *J Natl Cancer Inst.* 2002;94(2):94-101

4. Wei Q, Lee JE, Gershenwald JE, et al. Repair of UV light-induced DNA damage and risk of cutaneous malignant melanoma. *J Natl Cancer Inst.* 2003;95(4):308-315

5. Wang L-E, Xiong P, Strom SS, et al. In Vitro Sensitivity to Ultraviolet B Light and Skin Cancer Risk: A Case–Control Analysis. *JNCI: Journal of the National Cancer Institute.* 2005;97(24):1822-1831

6. Wang LE, Li C, Strom SS, et al. Repair capacity for UV light induced DNA damage associated with risk of nonmelanoma skin cancer and tumor progression. *Clin Cancer Res.* 2007;13(21):6532-6539

7. Wang LE, Li C, Xiong P, et al. 4-nitroquinoline-1-oxide-induced mutagen sensitivity and risk of cutaneous melanoma: a case-control analysis. *Melanoma Res.* 2016;26(2):181-187.PMC4948741

8. Spitz MR, Hsu TC, Wu X, Fueger JJ, Amos CI, Roth JA. Mutagen sensitivity as a biological marker of lung cancer risk in African Americans. *Cancer Epidemiol Biomarkers Prev.* 1995;4(2):99-103

9. Strom SS, Wu S, Sigurdson AJ, et al. Lung cancer, smoking patterns, and mutagen sensitivity in Mexican-Americans. *J Natl Cancer Inst Monogr.* 1995(18):29-33

10. Wu X, Delclos GL, Annegers JF, et al. A case-control study of wood dust exposure, mutagen sensitivity, and lung cancer risk. *Cancer Epidemiol Biomarkers Prev.* 1995;4(6):583-588

11. Wei Q, Cheng L, Hong WK, Spitz MR. Reduced DNA repair capacity in lung cancer patients. *Cancer Res.* 1996;56(18):4103-4107

12. Wei Q, Cheng L, Amos CI, et al. Repair of tobacco carcinogen-induced DNA adducts and lung cancer risk: a molecular epidemiologic study. *J Natl Cancer Inst.* 2000;92(21):1764-1772

13. Rajaee-Behbahani N, Schmezer P, Risch A, et al. Altered DNA repair capacity and bleomycin sensitivity as risk markers for non-small cell lung cancer. *Int J Cancer.* 2001;95(2):86-91

14. Spitz MR, Wu X, Wang Y, et al. Modulation of nucleotide excision repair capacity by XPD polymorphisms in lung cancer patients. *Cancer Res.* 2001;61(4):1354-1357

15. Shen H, Spitz MR, Qiao Y, et al. Smoking, DNA repair capacity and risk of nonsmall cell lung cancer. *International Journal of Cancer.* 2003;107(1):84-88

16. Spitz MR, Wei Q, Dong Q, Amos CI, Wu X. Genetic susceptibility to lung cancer: the role of DNA damage and repair. *Cancer Epidemiol Biomarkers Prev.* 2003;12(8):689-698

17. Paz-Elizur T, Krupsky M, Blumenstein S, Elinger D, Schechtman E, Livneh Z. DNA repair activity for oxidative damage and risk of lung cancer. *J Natl Cancer Inst.* 2003;95(17):1312-1319

18. Wang L, Wei Q, Shi Q, Guo Z, Qiao Y, Spitz MR. A modified host-cell reactivation assay to measure repair of alkylating DNA damage for assessing risk of lung adenocarcinoma. *Carcinogenesis.* 2007;28(7):1430-1436

19. Wu X, Lin J, Etzel CJ, et al. Interplay between mutagen sensitivity and epidemiological factors in modulatinglung cancer risk. *International Journal of Cancer.* 2007;120(12):2687-2695

20. El-Zein RA, Monroy CM, Cortes A, Spitz MR, Greisinger A, Etzel CJ. Rapid method for determination of DNA repair capacity in human peripheral blood lymphocytes amongst smokers. *BMC Cancer.* 2010;10:439.PMC2933626

21. Sigurdson AJ, Jones IM, Wei Q, et al. Prospective analysis of DNA damage and repair markers of lung cancer risk from the Prostate, Lung, Colorectal and Ovarian (PLCO) Cancer Screening Trial. *Carcinogenesis.* 2011;32(1):69-73.PMC3010173

22. Wang LE, Gorlova OY, Ying J, et al. Genome-wide association study reveals novel genetic determinants of DNA repair capacity in lung cancer. *Cancer Res.* 2013;73(1):256-264.PMC3537906

23. Leitner-Dagan Y, Sevilya Z, Pinchev M, et al. Enzymatic MPG DNA repair assays for two different oxidative DNA lesions reveal associations with increased lung cancer risk. *Carcinogenesis.* 2014;35(12):2763-2770.PMC4303808

24. Sevilya Z, Leitner-Dagan Y, Pinchev M, et al. Development of APE1 enzymatic DNA repair assays: low APE1 activity is associated with increase lung cancer risk. *Carcinogenesis.* 2015;36(9):982-991

25. Paz-Elizur T, Leitner-Dagan Y, Meyer KB, et al. DNA Repair Biomarker for Lung Cancer Risk and its Correlation With Airway Cells Gene Expression. *JNCI Cancer Spectr.* 2020;4(1):pkz067.PMC7012022

26. Zhao L, Chang DW, Gong Y, Eng C, Wu X. Measurement of DNA damage in peripheral blood by the γ-H2AX assay as predictor of colorectal cancer risk. *DNA Repair (Amst).* 2017;53:24-30

27. Schabath MB, Spitz MR, Grossman HB, et al. Genetic instability in bladder cancer assessed by the comet assay. *J Natl Cancer Inst.* 2003;95(7):540-547

28. Fernández MI, Gong Y, Ye Y, et al. γ-H2AX level in peripheral blood lymphocytes as a risk predictor for bladder cancer. *Carcinogenesis.* 2013;34(11):2543-2547.PMC3810842

29. Shao L, Lin J, Huang M, Ajani JA, Wu X. Predictors of esophageal cancer risk: assessment of susceptibility to DNA damage using comet assay. *Genes Chromosomes Cancer.* 2005;44(4):415-422

30. Xu E, Gong Y, Gu J, Jie L, Ajani JA, Wu X. Risk assessment of esophageal adenocarcinoma using γ-H2AX assay. *Cancer Epidemiol Biomarkers Prev.* 2013;22(10):1797-1804.PMC3824382

31. Cheng L, Eicher SA, Guo Z, Hong WK, Spitz MR, Wei Q. Reduced DNA repair capacity in head and neck cancer patients. *Cancer Epidemiol Biomarkers Prev.* 1998;7(6):465-468

32. Wang LE, Sturgis EM, Eicher SA, Spitz MR, Hong WK, Wei Q. Mutagen sensitivity to benzo(a)pyrene diol epoxide and the risk of squamous cell carcinoma of the head and neck. *Clin Cancer Res.* 1998;4(7):1773-1778

33. Xiong P, Hu Z, Li C, et al. In vitro benzo[a]pyrene diol epoxide-induced DNA damage and chromosomal aberrations in primary lymphocytes, smoking, and risk of squamous cell carcinoma of the head and neck. *Int J Cancer.* 2007;121(12):2735-2740

34. Wang L-E, Xiong P, Zhao H, Spitz MR, Sturgis EM, Wei Q. Chromosome instability and risk of squamous cell carcinomas of head and neck. *Cancer research.* 2008;68(11):4479-4485

35. Wang L-E, Hu Z, Sturgis EM, et al. Reduced DNA Repair Capacity for Removing Tobacco Carcinogen–Induced DNA Adducts Contributes to Risk of Head and Neck Cancer but not Tumor Characteristics. *Clinical Cancer Research.* 2010;16(2):764-774

36. Liu Z, Liu H, Gao F, Dahlstrom KR, Sturgis EM, Wei Q. Reduced DNA double-strand break repair capacity and risk of squamous cell carcinoma of the head and neck--A case-control study. *DNA repair.* 2016;40:18-26

37. Han P, Liu H, Shi Q, et al. Associations between expression levels of nucleotide excision repair proteins in lymphoblastoid cells and risk of squamous cell carcinoma of the head and neck. *Molecular carcinogenesis.* 2018;57(6):784-793

38. Wu X, Gu J, Patt Y, et al. Mutagen sensitivity as a susceptibility marker for human hepatocellular carcinoma. *Cancer Epidemiol Biomarkers Prev.* 1998;7(7):567-570

39. Xiong P, Bondy ML, Li D, et al. Sensitivity to benzo(a)pyrene diol-epoxide associated with risk of breast cancer in young women and modulation by glutathione S-transferase polymorphisms: a case-control study. *Cancer Res.* 2001;61(23):8465-8469

40. Smith TR, Miller MS, Lohman KK, Case LD, Hu JJ. DNA damage and breast cancer risk. *Carcinogenesis.* 2003;24(5):883-889

41. Shi Q, Wang LE, Bondy ML, Brewster A, Singletary SE, Wei Q. Reduced DNA repair of benzo[a]pyrene diol epoxide-induced adducts and common XPD polymorphisms in breast cancer patients. *Carcinogenesis.* 2004;25(9):1695-1700

42. Kennedy DO, Agrawal M, Shen J, et al. DNA repair capacity of lymphoblastoid cell lines from sisters discordant for breast cancer. *J Natl Cancer Inst.* 2005;97(2):127-132

43. Natarajan TG, Ganesan N, Carter-Nolan P, Tucker CA, Shields PG, Adams-Campbell LL. γ-Radiation-Induced Chromosomal Mutagen Sensitivity Is Associated with Breast Cancer Risk in African-American Women: Caffeine Modulates the Outcome of Mutagen Sensitivity Assay. *Cancer Epidemiology Biomarkers &amp; Prevention.* 2006;15(3):437-442

44. Bau D-T, Mau Y-C, Ding S-l, Wu P-E, Shen C-Y. DNA double-strand break repair capacity and risk of breast cancer. *Carcinogenesis.* 2007;28(8):1726-1730

45. Machella N, Terry MB, Zipprich J, et al. Double-strand breaks repair in lymphoblastoid cell lines from sisters discordant for breast cancer from the New York site of the BCFR. *Carcinogenesis.* 2008;29(7):1367-1372.PMC2899852

46. Wang LE, Han CH, Xiong P, et al. Gamma-ray-induced mutagen sensitivity and risk of sporadic breast cancer in young women: a case-control study. *Breast Cancer Res Treat.* 2012;132(3):1147-1155.PMC3523666

47. Matta J, Echenique M, Negron E, et al. The association of DNA Repair with breast cancer risk in women. A comparative observational study. *BMC Cancer.* 2012;12:490-490.PMC3572436

48. Shen J, Song R, Chow WH, Zhao H. Homologous recombination repair capacity in peripheral blood lymphocytes and breast cancer risk. *Carcinogenesis.* 2020;41(10):1363-1367.PMC7566366

49. Hu JJ, Hall MC, Grossman L, et al. Deficient nucleotide excision repair capacity enhances human prostate cancer risk. *Cancer Res.* 2004;64(3):1197-1201

50. Dong H, Jin X, Hu J, et al. High γ-Radiation Sensitivity Is Associated with Increased Gastric Cancer Risk in a Chinese Han Population: A Case-Control Analysis. *PLoS One.* 2012;7(8):e43625

51. Spitz MR, Fueger JJ, Beddingfield NA, et al. Chromosome sensitivity to bleomycin-induced mutagenesis, an independent risk factor for upper aerodigestive tract cancers. *Cancer Res.* 1989;49(16):4626-4628

52. Wu X, Gu J, Hong WK, et al. Benzo[a]pyrene diol epoxide and bleomycin sensitivity and susceptibility to cancer of upper aerodigestive tract. *J Natl Cancer Inst.* 1998;90(18):1393-1399

53. Bondy ML, Kyritsis AP, Gu J, et al. Mutagen sensitivity and risk of gliomas: a case-control analysis. *Cancer Res.* 1996;56(7):1484-1486
